# Telomere shortening associates with elevated insulin and nuchal fat accumulation

**DOI:** 10.1038/s41598-020-63916-6

**Published:** 2020-04-22

**Authors:** Harald Mangge, Markus Herrmann, Gunter Almer, Sieglinde Zelzer, Reinhard Moeller, Renate Horejsi, Wilfried Renner

**Affiliations:** 1 Clinical Institute of Medical and Chemical Laboratory Diagnostics, Medical University of Graz, Graz, Austria; 2 Otto Loewi Research Center (for Vascular Biology, Immunology and Inflammation), Medical University of Graz, Graz, Austria

**Keywords:** Biomarkers, Risk factors

## Abstract

Obesity and relative leucocyte telomere length (RTL) are both linked to accelerated aging and premature mortality. We examined if nuchal subcutaneous adipose tissue (SAT) thickness, a surrogate marker of central trunk-weighted obesity, is an independent predictor of RTL that provides information beyond BMI, metabolic and inflammatory markers. RTL and nuchal SAT thickness were determined in 362 participants of the STYJOBS/EDECTA study (STYrian Juvenile Obesity Study, Early DEteCTion of atherosclerosis), which included overweight individuals and matched eutrophic controls. Fasting plasma samples were used for the measurement of leptin, resistin, adiponectin, glucose, insulin, high-sensitivity C-reactive protein (hs-CRP), interleukin-6 (IL-6), liver enzymes, creatinine, cholesterol, high-density lipoprotein (HDL) cholesterol, low-density lipoprotein (LDL) cholesterol, oxidized LDL, triglycerides, homocysteine and uric acid. Furthermore, all participants underwent carotid artery ultrasound. Obese individuals had markedly higher body mass index (BMI), nuchal SAT thickness, hip and waist circumferences and carotid intima media thickness (IMT) than eutrophic controls. In addition, they showed typical biochemical abnormalities related to energy metabolism, systemic inflammation and liver function. RTL was inversely correlated with nuchal SAT thickness, IMT, hs-CRP, alkaline phosphatase, insulin, resistin, and leptin. Positive correlations were seen with homocysteine and creatinine. Stepwise linear regression analyses identified nuchal SAT thickness and insulin as the only significant predictors of RTL. In conclusion, nuchal SAT thickness is a robust predictor of RTL that provides information beyond traditional obesity-related metabolic and inflammatory biomarkers. This suggests an important role of fat depots at the neck for accelerated telomere shortening.

## Introduction

Telomeres are protective nucleoprotein structures at the end of all chromosomes that shorten with age. When telomeres become critically short, they induce apoptosis. Telomere length in circulating leucocytes is considered a surrogate marker of genomic aging in the entire body. In previous longitudinal studies, telomere length has firmly been established as a predictor of mortality risk. For example, in the Ludwigshafen Risk and Cardiovascular Health (LURIC) study, cardiovascular patients with the lowest relative leucocyte telomere length (RTL) had an 18% higher risk of mortality during an average follow-up period of 9.9 years when compared to all other participants^1^. Mons *et al*. analyzed 12,199 individuals from two population-based prospective cohort studies with an age range of 43–75 years. Participants in the first RTL quartile with the shortest telomeres had a 23% higher risk of dying from any cause than those in the fifth quintile with the longest telomeres^2^.

Solid knowledge about the factors that influence RTL is important for a potential clinical use of this promising marker and an appropriate interpretation of results. Lifestyle factors, such as physical activity, nutrition and stress have been suggested as potential modulators of telomere length^3–11^. For example, regular endurance exercise induces telomerase, an enzyme that can prolong telomeres, and thus prevent telomere shortening^4–7^. Nutritional factors also appear to be involved in telomere biology. Pusceddu *et al*. have shown that vitamin B6, B12 and homocysteine relate to RTL and mortality in cardiovascular patients^11^. In contrast, in longitudinal studies various diets or food supplements, considered generally healthy have shown little or no effect on telomere attrition^8,9^.

Obesity is a very common condition in Western societies that is associated with a broad range of metabolic alterations, elevated oxidative stress and chronic low-grade inflammation^10,12^. According to current concepts adipose tissue promotes the process of aging and drives the development of chronic diseases, such as type 2 diabetes (T2DM), non-alcoholic fatty liver disease (NAFLD), cancer, and cardiovascular diseases^12^. Based on the aforementioned observations it has been hypothesized that obesity accelerates telomere shortening. However, studies of leucocyte RTL in obese phenotypes yielded conflicting results, and the association between body mass index (BMI) and RTL during life time is still unclear^13–15^. A recent meta-analysis showed a converse relationship between BMI and RTL in cross sectional studies^16^. However, the authors of this meta-analysis claimed a lack of longitudinal studies with comprehensive controlling for age- and sex-specific confounders. Irrespective of an increased BMI, the location of fat accumulation appears to matter. In the past, three body types have been assigned based upon the distribution of body fat: android (apple shape), intermediate, and gynoid (pear shape). The apple shape has repeatedly been associated with an increased risk of diabetes, hypertension and hypercholesterolemia^17,18^.

Considering that the classification of body shape is rather subjective, alternative approaches, like waist-to-hip ratio (WHR) or weight-shape-index (WSI) have been explored. The analysis of subcutaneous adipose tissue (SAT) thickness at standardized anatomical points across the whole body using a recently developed optical lipometric device is a more objective and precise way to assess adiposity and fat distribution patterns^19^. Using this method, we have recently shown that RTL has a robust negative correlation with nuchal SAT thickness^20^. However, other circulating biomarkers that are commonly altered in obese subjects were not considered.

This raised the question if nuchal SAT remains an independent predictor of RTL when other metabolic and inflammatory biomarkers are included in a multivariate analysis. This gap of knowledge prompted us to analyze a broad range of additional obesity-related biomarkers and explore if nuchal SAT remains an independent predictor of RTL that provides information beyond these traditional biochemical analyses.

## Materials and Methods

### Study population

For the purpose of this study, we analyzed RTL and SAT distribution in all 362 participants of the STYJOBS/EDECTA study (STYrian Juvenile Obesity Study, Early DEteCTion of atherosclerosis) from whom DNA samples were available. The STYJOBS/EDECTA study cohort consists primarily of overweight/obese individuals and sex and age matched normal weight controls aged between 7–68 years. Participants were recruited between 2005–2014 through campaigning on local media, at our outpatient patient clinic at the Medical University Graz (Austria), at collaborating medical practitioners and in local schools. Overweight was defined as having a BMI > 90th and <97th percentile in underage individuals (≤18 years), and between>25 and <29.9 kg/m^2^ in adults (>18 years). Underage participants with a BMI > 97th percentile and adults with>30 kg/m^2^ were classified as obese. All participants underwent physical examination, fasting blood collection, carotid ultrasound and standardized lipometry. Moreover, height, weight, waist and hip circumference were assessed. Exclusion criteria were endocrine diseases (e.g. hypothyreosis, manifest type 2 diabetes), infectious, inflammatory or any other chronic diseases. Detailed information of STYJOBS is available at ClinicalTrials.gov (Identifier NCT00482924).

### Lipometry

SAT thickness was assessed at 15 anatomically well-defined body sites distributed from neck to calf on the left side of all participants. Lipometry was performed using an optical device (EU Patent No. 0516251). The sensor head of the lipometer, that is held perpendicular to the measurement site, consists of a set of light emitting diodes (λ = 660 nm, light intensity 3.000 mcd) and a photodetector, that measures the corresponding light intensities that are back scattered in the SAT. Calibration and evaluation were done using computed tomography (CT) as the reference method^21–23^.

### Analysis of telomere length

Genomic DNA was prepared from stored EDTA whole blood using a MagNA Pure instrument (Roche, Vienna, Austria). Purity and concentration of the extracted DNA were measured by UV photometry at 260 nm and 280 nm, respectively. DNA samples were diluted to a concentration of 10 ng/µl. RTL was measured by a qPCR assay developed by Cawthon^24^ with minor modifications. The assay quantified the ratio of average telomere length to a single-gene copy (RPLP0, previously denoted as “36B4”). All qPCR analyses were performed as triplicates in 96-well plates on a CFX96 Real-Time PCR Detection System (Bio-Rad Laboratories, Vienna, Austria). Total reaction volume was 25 µl. For the quantification of the single copy gene, forward primer 5′-CAGCAAGTGGGAAGGTGTAATCC-3′ and reverse primer 5′- CCCATTCTATCATCAACGGGTACAA-3′ were used. For the quantification of telomeres, forward primer 5′-CGGTTTGTTTGGGTTTGGGTTTGGGTTTGGGTTTGGGTT-3′ and reverse primer 5′-GGCTTGCCTTACCCTTACCCTTACCCTTACCCTTACCCT-3′ were used. Primers were purchased at Metabion GmbH (Martinsried, Germany). Each reaction contained 300 nmol/L forward primer, 300 nmol/L reverse primer, 40 ng DNA, 1x SYBR Green I Stain (Lonza Cologne GmbH, Cologne, Germany) and 2.5 µl PerfeCTa qPCR 10x SuperMix (Quanta, Beverly, USA). Thermal cycling profile was 10 min at 95 °C followed by 40 cycles of 15 s at 95 °C, 30 s at 54 °C and 30 s at 72 °C with fluorescence data collection. Each run included a standard curve made by dilutions of available HeLa-DNA (New England Biolabs, Frankfurt, Germany) to determine the quantity of targeted templates in each sample relative to the HeLa-DNA. RTL was calculated as the ratio of telomere quantity to single copy gene quantity. PCR efficiencies were 106% for telomere amplification and 94% for single copy gene amplification. Mean intra-assay coefficient of variability (CV) was 8.7% and plate-to-plate CV was 12.7%.

### Laboratory analysis

During the initial study visit, fasting blood samples were collected from all patients, and stored at −20 °C until analysis. Leptin, resistin and adiponectin were determined from human plasma by ELISAs from Biovendor Laboratory Medicine, Inc. (Brno, Czech Republic), oxidized low-density lipoprotein (LDL), and plasma insulin by commercially available ELISAs from Mercodia (Uppsala, Sweden). Intra- and inter-assay coefficients of variation for all ELISAs in our study were below 10%. HOMA-IR (homeostatic model assessment – insulin resistance) was calculated as the product of the fasting plasma insulin value (in micro units per ml) and the fasting plasma glucose value (in mmol/L), divided by 22.5^25^. High-sensitivity C-reactive protein, IL-6, liver transaminases [aspartate aminotransferase (AST), alanine aminotransferase (ALT), gamma-glutamyl transferase (gamma-GT)], alkaline phosphatase (ALP), creatinine, alkaline phosphatase, glucose, cholesterol, HDL-cholesterol, LDL-cholesterol, triglycerides, homocysteine (HCY) and uric acid were measured by commercial routine laboratory methods from Roche Diagnostics on a Cobas 8000 chemical analyzer (Roche Diagnostics Mannheim, Germany).

### Carotid artery ultrasound

The ultrasound protocol involved scanning of the bulbous near common carotid artery^26^ on both sides with a 12-to-5-MHz broad-band linear transducer on a HDI 5000 (ATL, Bothell, Washington, DC, USA). The same investigator performed all scans to identify the greatest wall thickness. Longitudinal images directed through the center of the artery were analyzed at each vessel site. Measurements were made from stored digital images by an experienced reader. The IMT was assessed at the far wall as the distance between the interface of the lumen and intimae, and the interface between the media and adventitia^26–28^. The maximal IMT was recorded at each of the vessel segments and averaged for the left and right sides. The lumen diameter was calculated as the inter-adventitial diameter minus twice the maximum far wall IMT. All diameters were measured during diastole to avoid image blurring due to systolic arterial wall motion, and to minimize the influence of blood pressure^28^.

### Ethics

STYJOBS/EDECTA was approved by the ethical committee of the Medical University of Graz (EK number 20–029 ex 08/09), and conducted in compliance with guidelines for human studies as described in the Helsinki Declaration of 1975, revised in 1996. Blood collection occurred after written informed consent by the participants. If the volunteers were under the age of 18 years, informed consent has been obtained from a parent and/or legal guardian.

### Statistics

All variables were checked for normal distribution using the Kolmogorov-Smirnov test. Mean and standard deviation (SD) were calculated for all normally distributed variables, whereas median and interquartile ranges (IQR) were determined for not normally distributed variables. Relationships between RTL and various indices of body composure were identified by Pearson correlation analyses. P-values < 0.05 were considered significant. In order to identify the strongest determinants of RTL we entered all variables that correlated significantly with RTL into a stepwise linear regression analysis. All statistical analyses were conducted using the PASW Statistics 24.0 package.

## Results

The 362 participants had a mean age of 34.2 + 14.0 (SD) years (range: 7.2–68years), a mean height of 170 + 10.6 (SD) cm (range: 126–197 cm) and a mean weight of 79.4 + 23.7 (SD) kg (range: 24–180 kg). Table 1 summarizes anthropometric, clinical and biochemical characteristics of all participants. BMI, hip and waist circumference as well as nuchal SAT thickness were markedly higher in obese individuals than in eutrophic controls. Obese participants also showed numerous biochemical abnormalities related to energy metabolism, systemic inflammation and liver function (Table 2).

**Table 1:** Anthropometric data, body measures, and subcutaneous SAT thickness (left body side).

	All participants N = 362 (male 156/female 206)	Normal weight N = 148 (male 55/female 93)	Overweight/Obese N = 214 (male 101/female 113)
	Mean ± SD/Median (IQR 25th–75th)	Mean ± SD/Median (IQR 25th–75th)	Mean ± SD/Median (IQR 25^th^–75^th^)
Age, yrs	35.9 (23.7–45.7)	34.8 (27.4–44.9)	37.5 (17.9–46.5)
Carotid IMT	0.06 (0.07–0.08)	0.066 ± 0.02	0.075 ± 0.02**
**Conventional body measures**			
Body mass index, kg/m²	26.0 (22.6–31.9)	21.9 (20.0–23.4)	30.5 (27.4–35.9) ^+++^
Waist circumference (cm)	88.0 (75–103)	74.0 (69.3–80.0)	100.0 (90.0–110.0)***
Hip circumference (cm)	101 (93–112)	91.8 ± 7.5	110.2 ± 14.0^+++^
Waist to hip ratio	0.87 (0.81–0.94)	0.81 (0.77–0.87)	0.91 (0.87–0.99) ^+++^
Waist to height ratio	0.52 (0.45–0.61)	0.44 (0.41–0.47)	0.59 (0.54–0.65) ^+++^
**Lipometry**			
Nuchal (mm)	6.1 (3.2–9.3)	2.7 (1.9–4.7)	7.9 (5.8–11.1)^+++^
Triceps (mm)	10.5 ± 4.8	8.6 ± 4.0	11.8 ± 4.9***
Biceps (mm)	8.2 ± 5.0	5.0 ± 3.3	10.5 ± 4.7***
Upper back (mm)	6.7 (4.3–9.6)	4.0 (2.4–5.3)	8.6 (6.8–11.0)^+++^
Front chest (mm)	9.4 (4.2–14.4)	3.9 (2.4–7.3)	12.9 (9.6–16.2) ^+++^
Lateral chest (mm)	10.6 (5.1–15.8)	4.7 (2.6–8.3)	14.6 (10.8–18.2)^+++^
Upper abdomen (mm)	12.3 (8.3–15.3)	7.6 (4.5–10.5)	14.1 (11.3–17.2) ^+++^
Lower abdomen (mm)	10.7 (8.0–14.1)	8.4 (5.2–10.8)	12.5 (10.1–15.4) ^+++^
Lower back (mm)	10.2 (7.7–12.5)	8.0 (5.7–10.5)	11.3 (9.4–14.1) ^+++^
Hip (mm)	11.3 (7.9–15.9)	8.5 (4.3–11.4)	13.8 (10.0–18.1) ^+++^
Front thigh (mm)	7.5 (5.3–10.0)	7.4 (4.5–9.6)	7.6 (5.7–10.1)
Lateral thigh (mm)	7.3 (4.2–9.6)	6.8 (3.6–9.2)	7.4 (4.6–9.9)
Rear thigh (mm)	5.8 (3.6–7.8)	5.4 (3.2–7.7)	5.8 (3.8–8.0)
Inner thigh (mm)	9.3 (7.0–11.7)	8.7 (6.9–10.9)	9.7 (7.1–12.3)
Calf (mm)	4.5 (2.9–6.4)	3.8 (2.3–5.5)	5.0 (3.3–7.0)

Data are presented as n (%) for categorical parameters and mean ± SD or median or median and IQRs for continuous parameters. ***p < 0.001, **p < 0.01, *p < 0.05 significances for t-test evaluation of normally distributed data; ^+++^p < 0.001, ^++^p < 0.01, ^+^p < 0.05 significances for not normally distributed data evaluated by Mann Whitney test.

**Table 2:** Blood biomarkers of the study cohort.

	All N = 362 (male 156/female 206)	Normal weight N = 148 (male 55/female 93)	Overweight/Obese N = 214 (male 101/female 113)
	Mean ± SD/Median (IQR 25th–75th)	Mean ± SD/Median (IQR 25th–75th)	Mean ± SD/Median (IQR 25^th^–75^th^)
Relative telomere length	0.52 (0.44–0.62)	0.53(0.45–0.64)	0.50 (0.43–0.59)^+^
hs-CRP, mg/L	1.8 (0.7–3.6)	0.79 (0.23–41.70)	2.7 (0.30–53.50)^+++^
Interleukin-6, pg/ml	2.0 (1.5–3.3)	1.6 (1.40–12.50)	2.4 (1.40–17.70)^+++^
Cholesterol, mmol/L	191 (161–220)	190 ± 40.9	198 ± 59.6
Triglyceride, mmol/L	91 (62–138)	68 (31–342)	118 (32–1028)^+++^
HDL-cholesterol, mmol/L	57 (44–72)	68 (57–80)	50 (40–62)^+++^
LDL-cholesterol, mmol/L	114 (94–139)	107 (85.7–135.7)	124 ± 43.4^++^
Oxidized LDL, mmol/L	54.0 (38.5–70.1)	43.9 (21.2–106.9)	60.7 (23.3–161.1)^+++^
Homocystein, µmol/L	11.4 (9.9–13.5)	11.4 (9.6–12.9)	12.0 0.81 ± 3.2
AST, U/L	26 (22–32)	24 (21–29)	29.7 ± 10.8^+++^
ALT, U/L	22 (17–34)	19 (16–24)	34.6 ± 22.6^+++^
GGT, U/L	21 (14–31)	17.5 (7–139)	24.0 (6–325)^+++^
CHE, U/L	8276 (7043–9809)	7369 ± 1415	9207 ± 1816***
Alkaline phosphatase, U/L	63 (50–82)	53 (19–334)	70 (11–342)^+++^
Creatinine, mg/dl	0.83 ± 0.17	0.86 ± 0.14	0.81 ± 0.19*
Uric acid, mg/dl	5.2 (4.3–6.1)	4.6 ± 1.17	5.7 ± 1.32***
Glucose, mg/dl	86 (80–93)	85 (80–90)	87 (80–96)^++^
Insulin, uE/ml	9.2 (5.5–14.8)	6.12 (0.24–24.20)	12.0 (2.06–58.66)^+++^
HOMA-IR	1.8 (1.1–3.1)	1.3 (0.06–4.79)	2.6 (0.29–13.08)^+++^
Resistin, µg/ml	4.7 (3.5–6.0)	4.4 (1.91–15.42)	4.8 (2.32–19.75)
Adiponectin, µg/ml	9.6 (6.7–12.8)	11.2 (0.01–36.4)	8.6 (1.71–32.55)^+++^
Leptin, ng/ml	13.6 (4.9–32.7)	5.1 (0.01–36.40)	27.8 (0.01–108.77)^+++^

Data are presented as n (%) for categorical parameters and mean ± SD or median or median and IQRs for continuous parameters. ***p < 0.001, **p < 0.01, *p < 0.05 significances for t-test evaluation of normally distributed data; ^+++^p < 0.001, ^++^p < 0.01, ^+^p < 0.05 significances for not normally distributed data evaluated by Mann Whitney test.

In the Pearson correlation analysis RTL was inversely correlated with nuchal SAT thickness, carotid IMT, hs-CRP, ALP, insulin, HOMA-IR, resistin, and leptin (Table 3). Positive correlations were seen with HCY and creatinine. The strongest correlation was found between RTL and leptin followed by nuchal SAT thickness.

**Table 3:** Pearson correlation analysis between RTL and related variables.

*r P* value		
Age, yrs	−0.030	0.512
Nuchal fat thickness, cm	−0.255	**<0.001**
Carotis IMT, cm	−0.198	**0.013**
Systolic BP, mmHg	−0.062	0.229
Diastolic BP, mmHg	−0.030	0.560
hs-CRP, mg/L	−0.139	**0.007**
Interleukin-6, pg/ml	−0.075	0.155
Cholesterol, mmol/L	0.016	0.764
Triglyceride, mmol/L	−0.039	0.456
HDL-cholesterol, mmol/L	0.068	0.192
LDL-cholesterol, mmol/L	0.013	0.812
Oxidized LDL, mmol/L	−0.070	0.185
Free fatty acids, mmol/L	−0.162	0.581
Homocystein, µmol/L	0.106	**0.044**
AST, U/L	0.022	0.667
ALT, U/L	0.010	0.843
GGT, U/L	0.047	0.366
CHE, U/L	−0.038	0.459
Alkaline phosphatase,U/L	−0.114	**0.028**
Creatinine, mg/dl	0.161	**0.002**
Uric acid, mg/dl	−0.020	0.702
Glucose, mg/dl	0.072	0.163
Insulin, uE/ml	−0.215	**<0.001**
HOMA-IR	−0.177	**0.001**
Resistin, µg/ml	−0.234	**<0.001**
Adiponectin, µg/ml	−0.049	0.353
Leptin, ng/ml	−0.271	**<0.001**

r = Pearson correlation coefficient; p = significance level, bold p-values are considered significant.

IMT = intima-media-thickness; hsCRP = high-sensitive C-reactive protein; IL-6 = interleukin-6; AST = aspartate transaminase; ALT = alanine transaminase; GGT = gamma-glutamyl-transpeptidase; CHE = cholinesterase.

Subsequently a stepwise linear regression analyses with RTL as the dependent variable was performed. As independent variables, we included sex, age and all biochemical analytes that were significantly correlated with RTL in the previous Pearson correlation analysis. This analysis identified nuchal fat thickness and insulin as the only significant predictors of RTL in the final model (Table 4).

**Table 4:** Linear stepwise regression analysis with RTL as dependent variable.

Final model	Standard β (95% CI)	p value
Nuchal fat thickness, mm	−0.400 (−0.017, −0.007)	**<0.001**
Insulin, uE/ml	−0.179 (−0.006, 0.000)	**0.048**

The following independent variables that were correlated with RTL were included: age, sex, nuchal fat thickness, carotis IMT, hs-CRP, homocysteine, AP, creatinine, insulin, resistin, leptin, and nuchal fat thickness.

## Discussion

In this study we provide evidence for a link between centralized SAT accumulation, disturbed glucose metabolism and genomic ageing. Nuchal SAT thickness appears to be a robust predictor of RTL that provides additional information beyond traditional obesity-related metabolic and inflammatory biomarkers. Fat depots at the neck and upper trunk seem to be particularly critical for accelerated shortening of telomeres. According to our observation, recent prospective studies showed that cardiovascular patients with an increased neck circumference have higher cardiovascular and all-cause mortality compared to those with a normal or low-neck circumference^29^. Our results further imply that a substantial part of the risk related to obesity is not captured by the measurement of traditional biomarkers, such as lipids, inflammatory markers or adipokines.

The link between nuchal SAT accumulation, plasma insulin levels and RTL relates a specific SAT distribution pattern to an altered glucose metabolism and accelerated genomic aging, indicated by short RTL. An association between obesity and telomere physiology has been reported before^13,30–36^. However, most previous studies used BMI to characterize individuals as overweight/obese or normal weight. A recently published meta-analysis of 87 observational studies including data from 146.114 subjects showed a significant inverse relationship between BMI and leucocyte telomere length, especially in young persons^37^. Mean telomere length decreased by 3.99 base pairs per unit increase in BMI^37^. However, on an individual basis the majority of the studies included show a trend, but failed to reach significance. Indirect evidence for an adverse effect of obesity on genomic aging comes from the observation that sustained weight loss 3–5 years after a gastric bypass operation is associated with an increase in RTL^38^. Even in childhood, obesity appears to link to short telomeres^39^. Otherwise, two recent studies challenged the clinical usefulness of RTL analysis in diabetes risk stratification. The first study investigated participants from the multiethnic Women’s Health Initiative and reported only limited clinical usability of RTL for diabetes risk stratification among postmenopausal women^40^. The second study analyzed RTL and glucose tolerance status in 205 mixed-ancestry South Africans and found no association of RTL with glycaemia^41^.

Body shape and fat distribution are important determinants of the health risks related to obesity (20,21). However, SAT distribution has not been considered systematically in previous investigations, especially in young people data are scarce. The strength of our study is the investigation of not only metabolic but also inflammatory parameters and RTL. Furthermore, an extensive noninvasive evaluation of the subcutaneous body fat distribution was performed, including young participants. According to current concepts, SAT is considered the least harmful adipose depot^42^. Nevertheless, when the SAT adipose cells become expanded (hypertrophic obesity), SAT become dysfunctional and drive the deposition of fat in ectopic depots, such as the liver and visceral organs. Increased hepatic and visceral fat reflects the inability of SAT to accommodate excess fat and lead to paracrine and endocrine effects that promote metabolic dysfunction^42^. The accumulation of visceral and nuchal fat depots often coincides in obese subjects and may represent an ‘overflow phenomenon’ where the storage capacity of SAT is exceeded so that fat has to be stored in ectopic depots^42^. If this process starts in the young and remains over time, the perdurability of “overflow” may create a more dangerous impact for serious obesity related clinical sequels. Risk algorithms including nuchal SAT thickness are missing because a simple method for the assessment of SAT distribution was not available in the past. The 15-point lipometry used here is a simple manageable tool that facilitates the assessment of SAT distribution. With the help of this method we have been able to identify “healthy obese” and “unhealthy lean” phenotypes in the STYJOBS cohort^43^. Moreover, we could show that nuchal SAT thickness is most predictive for other obesity-related biomarkers^44^.

Typically, obesity is accompanied by a broad range of metabolic alterations, elevated oxidative stress and chronic low-grade inflammation^12,45–51^. In previous studies, numerous blood biomarkers that address these pathomechanistic aspects have been linked to RTL. For example, significant associations with RTL have been reported for hs-CRP^52^, adiponectin^53^, and leptin^54^. Inflammation and oxidative stress of obesity are a pre-disease mechanisms for chronic diseases of ageing like cardiovascular disease, and cancer. The inflammatory cytokines TNF-α, IL-6 and IFN-γ may promote RTL shortening but direct influence lacks evidence and mechanisms remain ultimately unknown^55^. Interestingly, in the multivariate stepwise regression analysis of the present study insulin and nuchal SAT thickness but not hs-CRP or IL-6 were the only significant predictors of RTL. This argues for a strong influence of a disturbed glucose metabolism and central fat accumulation on RTL length. Previous case-control studies in patients with T2DM^56–58^ support this observation. A recent meta-analysis of 17 studies including 5575 diabetic patients and 6389 controls confirmed shorter RTL in T2DM patients in different geographic regions^58^. In type 1 diabetes (T1DM) this difference is much less pronounced, which highlights the potential role of insulin for telomere function. Circulating insulin is chronically elevated in T2DM until exhaustion of ß-cells in the later stages of the disease^59^. A recent 6-year prospective study of 67 T2DM patients revealed insulin use as a strong and significant predictor of accelerated telomere attrition in these patients. In a recent study of 44,168 individuals, investigating a new risk profile for all-cause mortality increased blood glucose levels had a strong influence to all-cause mortality^60^. The concept of insulin excess as a promotor of accelerated aging is also supported by a study in Asian Indians where Satishkumar *et al*. found an association between, insulin resistance, inflammation and cellular senescence^61^. These results have led to the hypothesis that RTL might be a biomarker of cellular senescence and predict CVD^62^. Supporting this hypothesis, Strazhesko I *et al*. showed that decreased RTL independently associates positively with atherosclerotic changes of the arterial wall, such as plaques number^62^. Primarily in 2010, Preis *et al*.^63^ showed in the Framingham Heart Study that increased neck circumference indicates a high risk for cardiovascular diseases. Recently, Caro *et al*.^64^ and Koppad *et al*.^65^ confirmed these observations. Our herein observed association between nuchal fat accumulations, decreased RTL and increased insulin levels may indicate the beginning of a pathological process leading to cardiovascular disease later in life. Several mechanism(s) linking RTL, insulin, and nuchal fat accumulation have been discussed. For example, hypo-methylation of the subtelomeric region has been proposed as a potential promotor of accelerated telomere shortening in T2DM^66^. However, this concept is not supported by another study of 418 Chinese T2DM patients where shorter leucocyte telomere length associated with LINE-1 hyper-methylation^56^.

Alternatively, high insulin levels may have direct damaging effects on telomeres and shelterin proteins. For example, Ras-proximate-1 or Ras-related protein 1(RAP1), a part of the shelterin complex, is a major regulator of energy metabolism^67^. In mice, inhibition of RAP 1 affects hepatic and adipose cell functions, resulting in disturbed glucose metabolism, insulin resistance, and obesity^68^. However, human studies exploring the link between obesity, insulin, RAP1 and RTL are lacking.

This study has limitations as follows. The lack of serial blood collections over a longer period prevents an evaluation of telomere dynamics in normal and overweight subjects. Furthermore, including low-weight subjects might have given important additional information. However, so far we were not able to recruit a relevant number of underweight/anorectic individuals. Another limitation is the method used for analysis of RTL. This qPCR method determines an average RTL value across chromosomes and cells, without considering differences between single chromosomes and cells. Moreover, we did not analyze glycemic statuses of participants, and individuals with an increased insulin concentration and participants with an abnormal HOMA index did not undergo an oral glucose tolerance test in order to prove insulin resistance. Finally yet importantly, the lipometry method used here describes SAT distribution but ignores visceral fat depots. Dual x-ray absorptiometry that can provide valuable information on body composition was unavailable for this study.

In conclusion, the relationship between nuchal SAT thickness, insulin and RTL observed in the present study suggests that central obesity and decreased insulin sensitivity are a fatal combination. Longitudinal studies are needed to explore the kinetics of RTL and glucose metabolism in normal and overweight subjects over time. These studies should keep track of future cardiovascular diseases and individual life expectancy.
